# The standard error of measurement is a more appropriate measure of quality for postgraduate medical assessments than is reliability: an analysis of MRCP(UK) examinations

**DOI:** 10.1186/1472-6920-10-40

**Published:** 2010-06-02

**Authors:** Jane Tighe, IC McManus, Neil G Dewhurst, Liliana Chis, John Mucklow

**Affiliations:** 1 MRCP(UK) Central Office, 11, St. Andrews Place, London NW1 4LE, UK; 2 Academic Centre for Medical Education and Research Department of Clinical, Educational and Health Psychology, University College London, London WC1E 6BT, UK

## Abstract

**Background:**

Cronbach's alpha is widely used as the preferred index of reliability for medical postgraduate examinations. A value of 0.8-0.9 is seen by providers and regulators alike as an adequate demonstration of acceptable reliability for any assessment. Of the other statistical parameters, Standard Error of Measurement (SEM) is mainly seen as useful only in determining the accuracy of a pass mark. However the alpha coefficient depends both on SEM and on the ability range (standard deviation, SD) of candidates taking an exam. This study investigated the extent to which the necessarily narrower ability range in candidates taking the second of the three part MRCP(UK) diploma examinations, biases assessment of reliability and SEM.

**Methods:**

a) The interrelationships of standard deviation (SD), SEM and reliability were investigated in a Monte Carlo simulation of 10,000 candidates taking a postgraduate examination. b) Reliability and SEM were studied in the MRCP(UK) Part 1 and Part 2 Written Examinations from 2002 to 2008. c) Reliability and SEM were studied in eight Specialty Certificate Examinations introduced in 2008-9.

**Results:**

The Monte Carlo simulation showed, as expected, that restricting the range of an assessment only to those who had already passed it, dramatically reduced the reliability but did not affect the SEM of a simulated assessment. The analysis of the MRCP(UK) Part 1 and Part 2 written examinations showed that the MRCP(UK) Part 2 written examination had a lower reliability than the Part 1 examination, but, despite that lower reliability, the Part 2 examination also had a *smaller *SEM (indicating a more accurate assessment). The Specialty Certificate Examinations had small Ns, and as a result, wide variability in their reliabilities, but SEMs were comparable with MRCP(UK) Part 2.

**Conclusions:**

An emphasis upon assessing the quality of assessments primarily in terms of reliability alone can produce a paradoxical and distorted picture, particularly in the situation where a narrower range of candidate ability is an inevitable consequence of being able to take a second part examination only after passing the first part examination. Reliability also shows problems when numbers of candidates in examinations are low and sampling error affects the range of candidate ability. SEM is not subject to such problems; it is therefore a better measure of the quality of an assessment and is recommended for routine use.

## Background

Any high-stakes examination should be as accurate, and hence as repeatable, as possible. The UK regulator, which used to be the Postgraduate Medical Education and Training Board (PMETB), repeatedly stated that reliability is of central importance in assessment [1-4]. On April 1st 2010, PMETB merged with the General Medical Council, the body responsible for the registration and regulation of UK doctors.

The usual measure of reliability in an assessment is Cronbach's coefficient alpha [5], with alpha taking values in the range 0 to 1, where 1 indicates perfect reliability and 0 indicates a test that is no better than marks awarded at random. A systematic review of the published literature on eleven postgraduate examinations in the US, UK, Canada and Israel [6] reported reliability coefficients, which typically were Cronbach's alpha, of between about 0.55 and 0.96, with a median of the order of 0.77. These examinations were heterogeneous in form using various methods from multiple-choice examinations to orals. A review of the reliability of the MRCP(UK) Part 1 Examination between 1984 and 2001, during which period the examination consisted of 300 true-false items with negative marking, showed that the mean reliability was 0.865 (SD 0.018; range = 0.83-0.89) [7]. Determining a lower acceptable value of alpha is not straightforward but the accepted minimum value for alpha in an examination has traditionally been 0.8, which it has been said that, "remains the benchmark below which an exam or elements within it should not fall. However, there is a consensus among medical educationalists that high stakes assessments ... should have a reliability of at least 0.9 (p.36) [3].

Although reliability is often presented as the sole statistic of importance in postgraduate examinations, the reasons for using it in isolation are not always made clear. Reliability depends both on Standard Error of Measurement (SEM) and on the ability range (standard deviation, SD) of candidates taking an assessment. The Standard Error of Measurement is a subtle and complex measure, and in particular there is a need to be careful in distinguishing SEM with the Standard Error of Estimation (SEE), the statistic that is appropriate when one wishes to estimate a candidate's true score from their actual score (for further details see Dudek [8], and also an introductory account by one of us (McManus IC: "The misinterpretation of the standard error of measurement in medical education: A brief primer on the problems, pitfalls and peculiarities", submitted). SEM is an adequate measure if one needs a general statistic for describing the likely accuracy of the score achieved by a randomly chosen candidate (but not for individual candidates at the extremes of the distribution of ability). Any individual candidate will, by definition, have a particular true score, and the SEM describes the likely range of actual scores such a candidate might achieve as a result of the unreliability of the assessment. A useful practical point to note is that the SEM in that sense is the same whether or not the candidate is of high, average or low ability, and there is no need to take regression to the mean into account, in contrast to the situation with SEE. The smaller the SEM, the more accurate are the assessments that are being made.

The usual calculation of SEM is straightforward and uses the formula:(1)

where SD is the standard deviation (the spread) of the marks of the candidates on the examination and the reliability is typically calculated as Cronbach's alpha, or some similar coefficient. However, it is worth pointing out that the calculation of SEM does not require a knowledge of reliability, and can be done from first principles (see Additional File 1); a worked example is provided for 5 candidates answering 10 binary items (see Additional File 2 and Additional File 3).

Equation 1 makes clear that, for any particular examination, the greater the reliability the smaller the SEM and hence the more accurate the examination, which is a desirable outcome. All other things being equal, high reliability is therefore generally to be desired as indicating a more accurate examination.

Something that is less often considered about equation 1 is that the SEM also depends crucially on the SD. Normally, little interest is taken in the SD, as for any particular set of examination marks it provides what appears to be a fixed constant, a mere description of the particular candidates who happened to be taking the exam, and whose only use is in calculating SEM. Holsgrove, however, points out that the reliability of an assessment can be improved not only by reducing the error variance, but that one "can also take steps to increase subject variance" [4]. Two separate approaches are possible: one method is to design the assessment so as to spread the candidates out, with the highest performers obtaining high marks and the poorest considerably lower ones. That method primarily uses items that are at the optimal level of difficulty for the candidates taking the exam. The second method is to increase the spread of ability levels in the candidates. To put it bluntly, if for whatever reason an assessment is taken by a greater number of very weak candidates, and perhaps also by a large number of very strong candidates, then SD will also be increased, and the reliability will apparently be higher. Halsgrove alludes to this phenomenon by saying, "Sometimes, especially in postgraduate examinations, we see a bimodal distribution of marks with UK graduates outperforming non-UK graduates and this can artificially inflate the reliability measurement". What is actually becoming clear in such an account is that a high reliability is not the *sine qua non *of an assessment. Such high values can be achieved in several ways that do not always reflect the true quality of the assessment, but rather are a function of who happens to be taking the assessment. Of course it must also be remembered that validity is the ultimate requirement of any assessment, although conventionally it is argued that validity cannot be achieved without a high reliability.

The principal interest of the present paper is in a situation in which SEM and reliability can apparently provide conflicting and seemingly paradoxical answers to the question of whether an assessment is acceptably accurate. The problem mainly arises in the situation where several examinations are taken sequentially, so that candidates are allowed to take a subsequent examination only when a previous one has been passed. That point is most easily shown by means of a simulation, after which we will then discuss actual data for the exams in question.

The paper will then go on to assess how the reliability and the SEM vary in relation to the MRCP(UK) Part 2 written examination, which has the particular feature, like many postgraduate examinations, that the second stage of the examination can be taken only by the highly selected candidates who have already passed the Part 1 examination. In effect, the candidates taking the Part 2 examination are similar to the candidates who passed the examination that we have simulated, and then went on to retake it. It would be expected, merely because of restriction of the ability range (and ignoring any changes in skills or abilities being assessed), that the reliability will be less in the Part 2 written examination than in the Part 1. Finally, we will look at the reliability of the recently introduced Specialty Certificate Examinations (SCEs), where numbers are extremely small, and reliability values can be highly variable.

### The MRCP(UK) examinations and Specialty Certificate Examinations

The MRCP(UK) is a three-part examination that provides summative assessment of knowledge requirements and clinical skills necessary for trainee physicians before undertaking higher training in internal medicine and/or a medical specialty. The MRCP(UK) Part 1 and Part 2 Written Examinations are criterion-referenced, single-version, machine-marked papers. The third part of the Examination is the practical assessment of clinical examination skills (PACES). Three diets (sittings) of each exam take place each year. The MRCP(UK) Part 2 Written Examination can be taken only following successful completion of the MRCP(UK) Part 1 Examination. The range of ability of candidates entering the MRCP(UK) Part 2 Examination is inevitably restricted in comparison with the MRCP(UK) Part 1 Examination, since only those who have passed the Part 1 Examination can enter the Part 2 Written Examination.

The formats of the Part 1 and Part 2 Examinations were substantially changed in 2002 and 2003. Since the 2003/3 diet for Part 1 and the 2002/3 diet for Part 2, each exam has consisted entirely of multiple-choice items that are all best-of-five format in Part 1, and are mostly best-of-five in Part 2, with a few questions which ask for the two best answers from a choice of ten. The Part 2 Written examination originally had about 150 test items per diet, in two separate three-hour papers (i.e. 75 items per paper). From the 2004/2 diet the examination was lengthened to a total of 180 scored items in two 3-hour papers (i.e. 90 items per paper). That change was driven in part by a concern that the reliability of the examination needed to be raised; and indeed, there was an increase in the reliability of the examination but the exam still failed consistently to achieve an alpha coefficient of 0.8, which at that time PMETB was suggesting was necessary for a high-stakes examination. From the 2005/3 diet of 2005, the MRCP(UK) Part 2 Written Examination was therefore increased to about 270 items on three 3-hour papers (i.e. about 90 questions per paper), with the exam held over two successive days. The longer format also had the advantage of comprehensive sampling from the curriculum, increasing the number of scored items and also of permitting the pre-testing of new items (which were not scored and were not taken by all candidates) without compromising the reliability.

Specialty Certificate Examinations were introduced in 2008 under the aegis of the Federation of Royal Colleges of Physicians of the UK, in collaboration with the various Specialist Societies, for eleven medical specialties (see http://www.mrcpuk.org/SCE/Pages/Home.aspx). The examinations all consist of two three-hour papers, each containing 100 best-of-five questions, administered by computer at a local test centre. Of necessity SCEs are taken by small numbers of candidates, being the final knowledge-based assessment for specialty trainees.

## Methods

Three separate studies were carried out.

a) A Monte Carlo analysis of the effects upon reliability and SEM of an examination being taken by all candidates, and then only those passing the first examination. A Monte Carlo analysis (which is named after the random numbers generated at roulette tables) generates large numbers of random numbers with particular characteristics, in order to assess the functioning of statistical methods, and is a standard computer-intensive technique within statistics. SPSS version 13.0 was used to generate normally distributed random numbers, which were treated as the true scores of candidates and the error scores of candidates taking the examination.

b) Reliability and SEM of the Part 1 and Part 2 Written examinations of the MRCP(UK) from 2002/3 to 2008/3. Data were analysed using SPSS version 13.0. The score on each assessment is calculated as the percentage of items answered correctly, with no correction for guessing. The standard deviation (SD), Cronbach's alpha coefficient, and the SEM were calculated using conventional methods. Because the examination mark is itself a percentage, the units of the SD and the SEMs are also expressed in percentage points.

c) Reliability and SEM of eight SCEs sat in 2008 and 2009, in eight different medical specialties. Analysis was as for the Part 1 and Part 2 examinations of MRCP(UK).

## Results

### The Monte Carlo simulation of successive examinations

**Figure 1:**
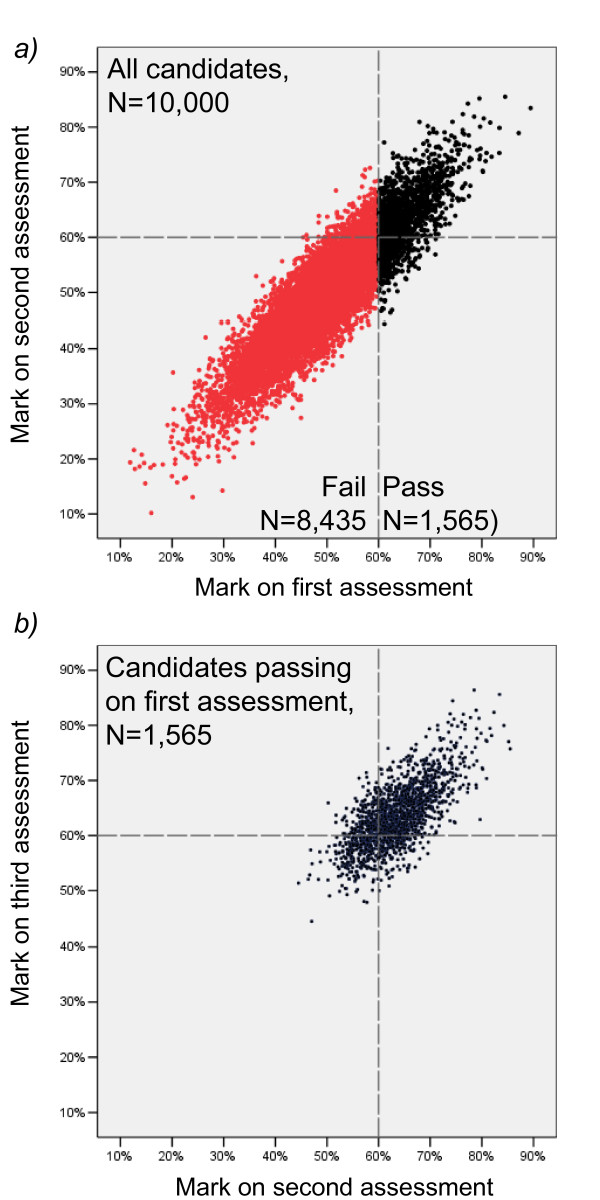
**In a Monte Carlo analysis, a simulated group of 10,000 candidates take an examination with a true mean of 50%, a true SD of 10%, a true reliability of 0.9, and a pass mark of 60%**. *Figure 1a *shows the candidates' marks on the first attempt (horizontal axis), with the pass mark shown as the vertical dashed grey line, the failing candidates shown in red and the passing candidates shown in black. All of the simulated candidates then take the examination again, and their marks on that second occasion are shown on the vertical axis, with the horizontal dashed line showing the same pass mark as was used on the first occasion. *Figure 1b *is restricted to the 1565 candidates who passed the examination on the first assessment, and shows the marks they obtained when they took the examination for the second time (horizontal axis), and then again on taking it for a third time (vertical axis). Once again the notional pass mark of 60% is indicated by the vertical and horizontal grey dashed lines.

The 'assessment' was taken by 10,000 randomly generated 'candidates', whose true scores were drawn from a normal distribution with a mean of 50% and a standard deviation of 10%. The sample size was intentionally large (although not unrealistically so for some national assessments) to ensure that sample statistics were close to their expected values (and for instance in the simulation, the mean true score for the randomly chosen 10,000 candidates was 50.007% and the SD was 9.954%). The true reliability of the assessment was set at 0.9, ensuring that the exam would meet PMETB's criterion for a reliable examination. Because this is only a simulation, we can also do what would not be possible in a real examination and require the 10,000 candidates to take the same examination twice under the same conditions. The correlation between the two marks was 0.897, very close to the expected value of 0.9, which is the reliability (see figure 1a).

The pass mark was set at 60%, and the 1565 individuals who pass on the first attempt (15.65%) are shown in figure 1a in black, while those who fail at the first attempt are shown in red. The horizontal axis shows the mark on the first occasion, and the vertical axis the mark on the second occasion. Even with a true reliability of 0.9 it can be seen that only 1107 individuals (11.07%) pass on both occasions, 458 individuals failing on the second occasion despite passing on the first, and 471 passing on the second occasion, despite failing on the first. Even with a reliability as high as 0.9, there are almost as many individuals who pass on one occasion and fail on the other (9.29%) as those who pass on both occasions (11.07%). Although 11% obtaining a different result on the two occasions may sound a high rate, it shows that even correlations [reliabilities] as high as 0.9 still have substantial amounts of measurement error associated with them, particularly around a cut mark.

The 1565 candidates who passed on the first occasion have already taken the exam on a second occasion, and now we can ask these candidates to take the exam for a third time. Figure 1b shows performance on the third occasion in relation to their performance on the second (and it should be emphasised that all of these candidates achieved a pass mark on the first occasion). It is clear that the black dots correspond to the same broad area of the scattergram as they did in figure 1a. However, and this is the key point, the correlation for the marks on the second and third occasion in these passing candidates is only 0.704. That value of 0.704 is therefore the reliability of the examination when it is administered only to candidates who have already passed the examination on the first attempt. It should be re-emphasised that this examination with reliability of 0.704 is for *precisely the same examination*, that earlier had a reliability of 0.897. Clearly the value of 0.704 is well below the oft quoted level of acceptability, whereas the value of 0.897 is acceptable. When used on one occasion this examination was acceptable and on another occasion the very same exam was unacceptable, a paradox that must cast doubt on the usefulness of reliability as an index. A key point is now apparent, one that is well recognised in the assessment literature: reliability is *not a property of an assessment*, but *a joint property of an assessment and the candidates who happen to take it*. Change the candidates and the reliability will also change.

The problem with reliability in the Monte Carlo simulation arises because the average SD of the marks on the second and third occasions shown in figure 1b is only 5.85%, compared with 10% in the entire set of candidates. It is an inevitable feature of the way that reliability is calculated, that if the range of marks is reduced then the reliability must go down. Reliability as a measure is therefore heavily dependent on the range of marks shown by a group of candidates. The larger the range of candidate ability the higher is the reliability, even when the assessment is identical.

What happens to the SEM? For the first assessment taken by all 10,000 candidates the SEM was 9.954 × √(1 - 0.905) = 3.07%. For the second and third assessments, taken only by the 1565 passing candidates, the SEM is 5.85 × √(1 - 0.704) = 3.18%. Within the limits of sampling variation, the SEM has not changed at all, despite being used on a much-restricted sample that is of much greater average ability than the total sample. That group is, of course, the group who can be conceptualised as going on to take a Part 2 exam, with a restricted range because of their greater ability. Even if that Part 2 assessment has the same measurement characteristics as the Part 1, it will necessarily have a lower reliability than the Part 1. While reliability is not therefore a good measure for testing the quality of a Part 2 examination, even when the examination is equivalent to the Part 1, the SEM *is *a good measure, maintaining its value even when the range is restricted. The Monte Carlo analysis carried out here has primarily been used for demonstrative purposes. It should however be emphasised that there is a standard correction for restriction of range which cannot also be applied. Using formula 10-11 on p.298 of Ghiselli et al [9], then with an unrestricted correlation of 0.9 and an unrestricted standard deviation of 10, then the effect of reducing the standard deviation to 5.85 is to reduce the expected correlation to 0.77, which is similar to the 0.704 actually found in our (single) example.

### The reliability of the MRCP(UK) Part 1 and Part 2 Written examinations

Table 1 shows the number of scored items on each examination, the alpha coefficient, the SD of candidate marks, and the SEM for each examination.

**Table 1:** Reliability of the MRCP(UK) Part 1 and Part 2 examinations.

	Part 1				Part 2			
**Diet**	**Number of scored items**	**Alpha**	**SD**	**SEM**	**Number of scored items**	**Alpha**	**SD**	**SEM**

2002/3	-	-	-	-	149	.79	7.67%	3.51%

2003/1	-	-	-	-	146	.76	7.43%	3.66%

2003/2	-	-	-	-	150	.73	6.94%	3.58%

2003/3	199	.89	9.23%	3.09%	152	.76	7.24%	3.52%

2004/1	200	.89	9.70%	3.10%	149	.75	7.10%	3.55%

2004/2	200	.89	10.46%	3.14%	177	.83	8.05%	3.28%

2004/3	200	.91	9.68%	3.14%	183	.78	6.94%	3.26%

2005/1	200	.89	10.67%	3.16%	181	.76	6.77%	3.30%

2005/2	200	.92	9.27%	3.08%	180	.80	7.33%	3.25%

2005/3	195	.90	10.19%	3.21%	253	.83	6.73%	2.78%

2006/1	194	.92	11.08%	3.23%	250	.81	6.46%	2.82%

2006/2	193	.90	10.09%	3.24%	251	.85	7.20%	2.75%

2006/3	195	.89	9.83%	3.27%	253	.82	6.52%	2.80%

2007/1	195	.92	11.49%	3.25%	249	.77	5.84%	2.83%

2007/2	195	.91	10.59%	3.25%	263	.84	6.89%	2.72%

2007/3	195	.92	11.51%	3.26%	262	.85	7.13%	2.76%

2008/1	184	.93	11.90%	3.15%	264	.82	6.52%	2.76%

2008/2	185	.91	11.13%	3.34%	266	.85	6.95%	2.73%

2008/3	185	.92	11.59%	3.28%	259	.84	6.99%	2.77%

**Mean (SD)** **All diets**	**194.7** **(5.57)**	**.907** **(.014)**	**10.53%** **(0.68%)**	**3.20%** **(.08%)**	**212.5** **(49.7)**	**.802** **(.039)**	**6.98%** **(0.48%)**	**3.09%** **(0.36%)**

**Mean (SD)** **2005/3 to 2008/3**	**191.6** **(4.84)**	**.912** **(.012)**	**10.94%** **(0.72%)**	**3.25%** **(0.05%)**	**257.0** **(6.46)**	**.828** **(.025)**	**6.72%** **(0.40%)**	**2.77%** **(0.04%)**

The Part 1 papers consist entirely of Best-of-Five questions. The Part 2 papers are mostly Best-of-Five questions, with two or three >Several-from-Many (questions in each diet. Negative marking is not used in either examination.

The reliability of the Part 2 examination (mean = 0.802) is consistently lower than that of the Part 1 examination (mean = 0.907), and the SD of the candidate marks is also consistently higher in the Part 1 examination (mean = 10.53%) than in the Part 2 examination (mean = 6.98%). The number of items in the Part 1 examination remained stable across the diets, as did the SD and the reliability, so that the SEM also remained at much the same value across the diets. Although the SD of candidate marks remained stable in the Part 2 examination, there was a substantial increase in the number of test items in the Part 2 examination starting with the 2005/3 diet, and from 2005/3 onwards there was therefore both an increase in the reliability of the Part 2 examination and a decrease in the SEM.

A striking thing about the results in table 1 is that although from 2005/3 onwards the SEM for the Part 2 examination (mean = 2.77%) was *lower *than that for the Part 1 examination (mean = 3.20%) (and the scales of the two examinations are on comparable percentage scales), the reliability of the Part 2 examination (mean = 0.828) *was also lower than that for the Part 1 examination *(mean = 0.907).

### The reliability of the Specialty Certificate Examinations

Table 2 summarises the results for the first eight Specialty Certificate Examinations. The average number of candidates was small, with a range from 6 to 39. Alpha coefficients on average were similar to those in the Part 2 examination (mean = 0.829), although the one very low alpha of 0.48, meant that the median of 0.87 was somewhat higher than the mean, being midway between the reliability of the Part 1 and Part 2 examinations. Standard deviations of candidate scores also showed large variation (3.97% to 12.13%), and when that was taken into account there was little variation in the SEM (range = 2.52% to 3.03%), with the mean of 2.87% being similar to the 2.77% found in the Part 2 examination, and substantially lower than the 3.25% found in the Part 1 examination.

**Table 2:** Reliability of the first eight Specialty Certificate Examinations.

Year	Specialty	Candidates	Number of scored items	Alpha	SD	SEM
2008	Gastroenterology	8	200	.84	7.00%	2.80%

2009	Dermatology	39	200	.88	7.27%	2.52%

2009	Endocrinology and Diabetes	39	200	.89	9.03%	2.99%

2009	Geriatric Medicine	15	200	.48	3.97%	2.86%

2009	Infectious Diseases	6	200	.94	12.13%	2.97%

2009	Neurology	25	200	.89	9.13%	3.03%

2009	Nephrology	33	200	.86	7.80%	2.92%

2009	Respiratory Medicine	25	200	.85	7.47%	2.89%

**Mean** **(SD)**	**All SCEs (n = 8)**	**23.8** **(13.1)**	**200** **(0)**	**.829** **(.144)**	**7.97%** **(2.31%)**	**2.87%** **(.16%)**

*Mean* *(SD)*	*MRCP (UK) Pt1* *2005/3 to 2008/3*		*191.6* *(4.84)*	*.912* *(.012)*	*10.94%* *(0.72%)*	*3.25%* *(0.05%)*

*Mean* *(SD)*	*MRCP(UK) Pt2* *2005/3 to 2008/3*		*257.0* *(6.46)*	*.828* *(.025)*	*6.72%* *(0.40%)*	*2.77%* *(0.04%)*

Results are scored as percentage of answers correct, and therefore are directly comparable with value shown in Table 1.

## Discussion

It is important that the quality of postgraduate medical examinations is assessed and maintained; important for candidates, for whom the examinations are a large investment of time and money; for the profession, for whom the examinations are central not only to maintaining standards, but also, in the long run, to increasing standards, by ensuring adequate knowledge and skills; and, most important of all, for patients, whose successful and effective treatment depends on the knowledge and skills of their medical practitioners.

The reliability of an examination provides useful information about its performance (and it is self-evident that an examination with a very low reliability is unlikely to be a good or an effective examination, to the point where zero reliability means that the marks from an examination are no more effective than are random numbers at distinguishing between candidates). Having said that, the mere fact that an examination has a high reliability does not ensure that it is necessarily functioning effectively, because the reliability is heavily dependent upon the ability range of the candidates who are taking it. As has already been seen:

i. The very same exam can apparently drop its reliability dramatically if it is retaken but only by those who have already passed it;

ii. The reliability can be artificially inflated by encouraging very weak candidates to take it, thereby increasing the SD of the marks;

iii. It is almost inevitable where successive examinations are taken, as with the Part 2 Written examination of MRCP(UK) being taken after Part 1, that the SD will necessarily be lower (only able candidates passing Part 1), and that the reliability of a second examination will usually be lower than the first examination.

iv. When examinations have very small numbers of candidates, as with the SCEs, there is a greater risk that the reliability will be distorted by an unusually high or low spread of candidate abilities (and in table 2, the highest reliability of 0.94 was for the examination where candidates has an SD of 12.1%, and the lowest reliability of 0.48 was for the examination where candidates had an SD of only 3.97%).

Reliability can always be increased by making an assessment progressively longer, thereby increasing the number of examination items, although that is expensive in time, effort and opportunity cost. The relationship between examination length and reliability is formalised in the Spearman-Brown formula:

The Spearman-Brown formula shows not only that in order to increase the reliability of an examination it has to be made much longer, but it also shows by how much longer. With 260 items, the reliability of the MRCP(UK) Part 2 Written examination is about 0.83. The formula shows that, to produce a reliability of 0.9, the examination would need about 450 items. The present 260 item examination takes one and a half days to administer, and therefore a 450 item assessment would last two and a half days. However admirable a high reliability may be, it seems unlikely that candidates or examiners would tolerate an examination of that length (particularly as it would be proportionately more expensive and time-consuming to set).

The Standard Error of Measurement has many advantages over reliability, not least that it is almost entirely a function of the collective performance of the items being used in an examinationDISCUSSION, rather than being a function *both *of those items *and *the ability range of the candidates. In effect, therefore, the SEM can be seen as a fundamental property of the ruler itself, rather than of a ruler in relation to the heights of the people who are being measured. As Weiss and Davison [10] have pointed out, it is only psychometrics that shows a "pre-occupation" with reliability coefficients, other sciences being much more concerned with error of measurement directly. This is not the place to discuss the interpretation of SEM, which depends upon the context in which it is being used, but interested readers are particularly referred to the clear and important paper of Dudek [8].

Although PMETB implies in some parts of its literature that SEM is important, there is much ambivalence about that position. For instance, the 2007 *Guide to Good Practice *comments that:

"In terms of assessment development, the SEM can help in identifying individual assessments that need to be improved, *though the reliability coefficient is more important in this regard*. The main use of the SEM, however, is to enable the proper identification of the borderline trainees - those whom the examination has not been able to confidently place on one side or the other of the pass mark. (p.37 (our emphasis) [2]

PMETB suggests therefore that reliability is particularly important, and SEM has but a subsidiary role in identifying those trainees who are borderline. That logic though is surely flawed. The most important thing in any high-stakes qualifying examination is the accuracy of the pass mark, which is determined by the SEM (and this, as the simulation has shown, is independent of the reliability and the SD of the candidates). If the reliability of an examination is increased merely by including more very weak and very strong candidates, that will appear to be effective in producing a better examination, even though there has been no change in the SEM, and hence no change in the accuracy of classification at the cutting point. As the simulation showed, for the highly selected sub-group the SEM remained a rational and appropriate quality indicator even though the reliability plummeted.

A problem with all arbitrary targets is that they encourage behaviour that concentrates on achieving the target, rather than on the real purpose behind the targets. By continually emphasising reliabilities of 0.8 or even 0.9, regulators run the risk that those who run postgraduate examinations will be distracted into chasing after those numbers. In a recent article entitled, "The seven deadly sins of assessment", "Lust", was classified by Tweed and Wilkinson [11] as, "the desire to improve the reliability coefficient to the point of redundancy and trivialisation, whilst ignoring the other important attributes of assessment" (p.165).

That the MRCP(UK) Part 2 Written examination has a lower reliability than the Part 1 examination tells one little of use about the assessment, and neither does the fact that the reliability of the SCE in Infectious Diseases is nearly twice that of the SCE in Geriatric Medicine. What is clear is that there are good statistical reasons why reliability will be lower when there is a narrower ability range in the candidates, and that in all of these cases the accuracy of measurement is comparable and more than adequate to the task (perhaps even too good, given the practical constraint that the Part 2 examination has to take place over two days rather than one).

It should also be remembered that, even with examinations of fixed length taken by candidates with a fixed range of ability, the SEM can still be improved by using items that perform better (in effect, have a greater item-total correlation in terms of classical test theory, or a greater discrimination in terms of item-response theory). The result will be an examination that is genuinely better at measuring ability, rather than one that merely pushes up reliability by other means of little real consequence. Of course, in practical terms, there is a finite limit on improvements in item quality.

## Conclusions

Standard error of measurement is a better measure of the quality of an assessment than is reliability, particularly when the ability range of the candidates must necessarily be restricted, as is the case of examinations such as the MRCP(UK) Part 2 Written, for which a condition of taking it is that the Part 1 examination has already been passed, or for small examinations in which sampling error alone can result in wide variation in average candidate ability. The problems of an undue emphasis upon reliability can readily be seen when simulations are used to model assessment processes.

## Abbreviations

GMC: General Medical Council; MRCP(UK): Membership of the Royal Colleges of Physicians (United Kingdom); PMETB: Postgraduate Medical Education and Training Board; SCE: Specialty Certificate Examination; SD: Standard Deviation; SEE: Standard Error of Estimation; SEM: Standard Error of Measurement

## Competing interests

The authors declare that they have no competing interests.

## Authors' contributions

JT initiated the study and with NGD wrote the first draft, LC was responsible for data analysis, JM helped with the analysis of the SCEs, ICM carried out the Monte Carlo analysis and wrote the second draft, and all authors contributed to the writing of the final manuscript. All authors read and approved the final manuscript.

## Pre-publication history

The pre-publication history for this paper can be accessed here:

http://www.biomedcentral.com/1472-6920/10/40/prepub

## Supplementary Material

Additional file 1 **The relationship between reliability, standard error of measurement and the standard deviation of marks**. Click here for file

Additional file 2 **An example calculating reliability and standard error of measurement for a small sample from raw data**. Click here for file

Additional file 3 **Spreadsheet for calculating reliability and SEM from a very sample**. Click here for file
