# Supplementary material for: The standard error of measurement is a more appropriate measure of quality for postgraduate medical assessments than is reliability: an analysis of MRCP(UK) examinations
Source: BMC Med Educ. 2010 Jun 2;10:40. doi: 10.1186/1472-6920-10-40 (PMC2893515; doi:10.1186/1472-6920-10-40)
Supplement: Additional file 1 — The relationship between reliability, standard error of measurement and the standard deviation of marks. [file 1472-6920-10-40-S1.PDF]

Additional file 1: The relationship between reliability, standard error of measurement and the standard deviation of marks.

The standard error of measurement (SEM) is conventionally calculated from the standard deviation of the scores in the population (σ_x) and the reliability of the scale (r_{xx}), using the formula:

$$SEM = \sigma_x \sqrt{1 - r_{xx}}$$

The reliability of a scale, r_{xx} , can be calculated in several ways, of which the most general is Cronbach's coefficient alpha (α), which is calculated using the formula:

$$\alpha = r_{xx} = \frac{k}{k-1}\left(1 - \frac{\Sigma\sigma_{x_i}^2}{\sigma_x^2}\right)$$

k is the number of items in the test, σ^2_x is the variance of the scores in the population, and $\Sigma\sigma^2_{xi}$ is the sum of the variances of each of the *k* test items (where *i* = 1, *k*)[1].

The two equations can be combined, so that:

$$SEM = \sigma_x \sqrt{1 - \frac{k}{k-1}\left(1 - \frac{\Sigma\sigma_{x_i}^2}{\sigma_x^2}\right)}$$

Notice that for a large number of test items, k≅(k-1), and hence k/(k-1) ≅1. As result the equation can be simplified to provide an approximation to SEM, which, to avoid confusion is called SEM* :

$$SEM^* = \sigma_x \sqrt{1 - \frac{k}{k-1}\left(1 - \frac{\Sigma\sigma_{x_i}^2}{\sigma_x^2}\right)} \cong \sigma_x \sqrt{1 - 1\cdot\left(1 - \frac{\Sigma\sigma_{x_i}^2}{\sigma_x^2}\right)} = \sigma_x \sqrt{\left(\frac{\Sigma\sigma_{x_i}^2}{\sigma_x^2}\right)} = \sqrt{\Sigma\sigma_{x_i}^2}$$

Notice that when *k* is large, and hence SEM* is a good approximation, the standard error of measurement, SEM, depends only on the summed item variances, and is independent of the overall variance of the scores of the candidates.

When *k* is small, an exact calculation of SEM can still be derived from the standard formula, since by rearranging, one obtains the following formula, which we call SEM_a.

$$SEM_a = \sigma_x \sqrt{1 - \frac{k}{k-1}\left(1 - \frac{\Sigma\sigma_{x_i}^2}{\sigma_x^2}\right)} = \sqrt{\frac{k.\Sigma\sigma_{x_i}^2}{k-1} - \frac{\sigma_x^2}{k-1}}$$

[1] Note that in the formula for alpha, the values of the standard deviation and the variance use the formulae designed for samples (i.e. divided by (n-1)) rather than those used for populations (i.e. divided by n).

In SEM_a it can be seen that as k becomes larger, so $k/(k-1)$ becomes closer to one, and the contribution of the variance of the scores in the population (σ^2_x) becomes progressively smaller, until with infinitely large k the equation is that for SEM^*.

An interesting corollary of the alternate formula, SEM_a, is that it can be used even in the unusual situation when the variance of the candidate scores is zero, which can occur with small numbers of candidates if it just so happens that all candidates get identical total marks, albeit from answering different combinations of questions correctly. Since $\sigma^2_x = 0$, alpha cannot be calculated from the standard formula, since there would be division by zero. However, in SEM_a, the component $\sigma^2_x /(k-1)$ becomes zero, which is valid, and the rest of the formula still applies, allowing an exact standard error of measurement to be calculated in the absence of a measure of reliability.
