# Supplementary material for: The standard error of measurement is a more appropriate measure of quality for postgraduate medical assessments than is reliability: an analysis of MRCP(UK) examinations
Source: BMC Med Educ. 2010 Jun 2;10:40. doi: 10.1186/1472-6920-10-40 (PMC2893515; doi:10.1186/1472-6920-10-40)
Supplement: Additional file 2 — An example calculating reliability and standard error of measurement for a small sample from raw data. [file 1472-6920-10-40-S2.PDF]

Additional file 2: An example calculating reliability and standard error of measurement for a small sample from raw data.

Although Cronbach's alpha and SEM are usually calculated using standard statistical packages such as SPSS, it is straightforward to calculate both from first principles using a spreadsheet program such as Excel (and the example shown below can be found as an Additional Material File and at http://preview.tinyurl.com/ybfhl7u). Here the calculation is carried out for a very small number of subjects (5) taking a very small test with only 10 binary items. The advantage is that the ways the numbers are combined, and hence their origins, are more easily visible.

In this example the scores of each of the five persons are shown on the rows, with the ten test items in the columns, each being scored as right or wrong.

Examination items (1=correct; 0 = incorrect)

		1	2	3	4	5	6	7	8	9	10	Total
Persons	1	0	1	0	1	1	1	0	0	1	0	5
	2	1	0	0	0	0	0	0	1	1	0	3
	3	1	0	0	1	1	1	0	1	1	1	7
	4	1	0	1	0	0	1	0	1	0	1	5
	5	1	1	1	1	1	1	0	1	1	1	9
Item variances		.20	.30	.30	.30	.30	.20	.00	.20	.20	.30	

Statistics for persons:

Mean	5.80
SD = σ_x	**2.28**
Variance = σ^2_x	5.20

Sum of item variances = .20 + .30 +.30 ....+.30 = **2.30** = $\Sigma\sigma^2_{xi}$

Calculation of Cronbach's alpha:

$$\alpha = r_{xx} = \frac{k}{k-1}\left(1-\frac{\Sigma\sigma^2_{x_i}}{\sigma^2_x}\right) = \frac{10}{9}\left(1-\frac{2.30}{2.28^2}\right) = 0.620$$

Calculation of SEM using standard formula:

$$SEM = \sigma_x\sqrt{1-r_{xx}} = 2.280\sqrt{1-0.620} = 1.41$$

Calculation of SEM using alternate formula[1]:

$$SEM_a = \sqrt{\frac{k.\Sigma\sigma^2_{x_i}}{k-1} - \frac{\sigma^2_x}{k-1}} = \sqrt{\frac{10\times 2.30}{9} - \frac{5.20}{9}} = 1.41$$

Calculation of approximate SEM* which does not require knowledge of reliability:

$$SEM^* \cong \sqrt{\Sigma\sigma^2_{x_i}} = \sqrt{2.300} = 1.52$$

Note that SEM and SEM* differ primarily because *k* is small. For a typical exam with k>100, there would be little difference between them.

A peculiar feature of the recent PMETB document on assessing reliability in examinations

[1] The values for SEM and SEMa differ in their last decimal place if calculated to three decimal places, due to rounding error, alpha actually being .619658.

with small numbers of candidates (Postgraduate Medical Education and Training Board, 2009) is the claim that "Without the reliability coefficient we cannot calculate the Standard Error of Measurement (SEM)" (p.5), and the statement later on the same page that, "Reliability measures ... are impossible for small cohorts", because, from the same page, "we need a sample of at least a certain minimum size in order to be able to make the calculation [of the reliability]". The example shown above suggests that none of these statements are accurate. Firstly, a good approximation to SEM can be calculated without knowing the reliability, and secondly, the reliability can be calculated for a sample even as small as the five shown here. In fact the calculations continue to work even when there are only two subjects (but not, of course, one) and two items (but not one), as long as variance remains non-zero. Such estimates of the reliability and the SEM would not themselves be very reliable, but that is another matter. If they were needed, confidence intervals could be calculated either by bootstrapping, or by various approximations that have been proposed (see Liu and Weng (2009) for an overview of the literature on calculating standard errors of coefficient alpha in various contexts).

Reference List

Liu, H.-Y. & Weng, L.-J. (2009). An effect size index for comparing two independent alpha coefficients. *British Journal of Mathematical and Statistical Psychology, 62,* 385-400.

Postgraduate Medical Education and Training Board (2009). *Reliability issues in the assessment of small cohorts (Guidance 09/1)*. London: PMETB (www.pmetb.org.uk).
